# Association of LDL to HDL ratio with new-onset atrial fibrillation after on-pump coronary artery bypass graft surgery

**DOI:** 10.1186/s12872-022-03016-7

**Published:** 2022-12-23

**Authors:** Ming-Huan Yu, Ren-Jian-Zhi Zhang, Xin-Yi Yu, Jian-Wei Shi, Zhi-Gang Liu

**Affiliations:** Department of Cardiovascular Surgery, TEDA International Cardiovascular Hospital, Chinese Academy of Medical Sciences and Graduate School of Peking Union Medical College, 61, Third Avenue, TEDA, Tianjin, China

**Keywords:** Coronary artery bypass graft, Postoperative atrial fibrillation, LDL/HDL ratio

## Abstract

**Objective:**

This study aims to analyze the association between preoperative LDL/HDL ratio and new-onset atrial fibrillation (AF) after on-pump coronary artery bypass grafting (on-pump CABG), evaluate the clinic value of preoperative LDL/HDL ratio to identify postoperative rhythm.

**Methods:**

A retrospective study of consecutive patients (*n* = 2052) who underwent on-pump CABG at TEDA International Cardiovascular Hospital (Tianjin, China), from June 1, 2020, to December 30, 2021, was conducted. The association between preoperative LDL/HDL and new-onset POAF was analyzed by Lowess curve and univariate logistic regression. The receiver operating characteristic curve (ROC) and area under the curve (AUC) were used to evaluate the identification capacity of preoperative LDL/HDL level for new-onset POAF.

**Results:**

In studied populations, the incidence of new-onset POAF was about 29.24%. The lowess curve showed that the association between preoperative LDL/HDL ratio and POAF after on-pump CABG was similar to a linear relationship. With the increasement of preoperative LDL/HDL ratio, the incidence of POAF increased simultaneously. ROC analysis showed that preoperative LDL/HDL ratio could identify postoperative arrhythmia after on-pump CABG (AUC = 0.569,95% CI = 0.529–0.608, *P* = 0.006) among female patients, the best preoperative LDL/HDL ratio cutoff of 2.11, which was considered a predictive factor of incident POAF, showed a sensitivity of 83.60% (95% CI = 0.775–0.886) and a specificity of 30.02% (95% CI = 0.257–0.346).

**Conclusion:**

Preoperative LDL/HDL ratio is associated with new-onset POAF, but there is a difference in different sex. Preoperative LDL/HDL level can help to identify postoperative rhythm in females.

## Introduction

Coronary atherosclerotic heart disease is a common cardiovascular disease, which is also the leading cause affecting public health and contributing to death [1]. The main treatment for coronary disease consists of drug administration, percutaneous coronary intervention (PCI), and coronary artery bypass grafting (CABG). However, atrial fibrillation (AF) is one of the common complications after CABG, with prevalence varying from 20 to 30% [2–4]. Postoperative AF (POAF) is associated with high stroke risk and in-hospital or long-term mortality, prolonging hospitalization.

Lipid level is associated with the incidence of AF [5, 6]. Low-density lipoprotein (LDL) and high-density lipoprotein (HDL) are two vital lipoproteins in serum. The association between LDL/HDL ratio and cardiovascular diseases has been demonstrated in several studies [7–9]. In addition, the preoperative LDL/HDL ratio is also correlated with graft patency after cardiac surgery [10]. However, there are inconsistencies in the relationship between LDL or HDL and POAF [6, 11, 12], and rare studies evidence the association between LDL/HDL ratio and POAF.

CABG can be operated on-pump or off-pump with a different incidence of POAF. The incidence of new-onset POAF after on-pump CABG is higher compared with off-pump CABG [13]. In the previous studies, the serum LDL and HDL level changed after on-pump CABG and are related to postoperative systematic inflammation [14, 15]. This study aims to analyze the association between preoperative LDL/HDL ratio and new-onset POAF after on-pump CABG and explore the potency of LDL/HDL ratio to predict new-onset POAF, providing a theoretical basis for the prediction and prevention of complications after on-pump CABG.

## Method

### Patient

This study included patients who underwent on-pump CABG at TEDA International Cardiovascular Hospital (Tianjin, China), from June 1, 2020, through December 30, 2021. Inclusion criteria were (1) No history of cardiac surgery, admission for the first time, and receiving on-pump CABG solely. (2) Grafts were the Internal Mammary artery and(or) great saphenous vein (3) Routine preoperative electrocardiogram was sinus rhythm, and no preoperative antiarrhythmic drugs were taken (except for β-receptor blocker). Exclusion criteria were (1) Preoperative combination of aortic dissection (2) Permanent pacemaker implantation. (3) AF history. (4) left atrial appendage thrombus diagnosed by preoperative echocardiogram. This study was approved by the Ethics Committee (Internal Review Board) of TEDA International Cardiovascular Hospital. All the procedures performed in this study involving human participants were conducted by the Declaration of Helsinki (as revised in 2013). All data collection was done anonymously. The requirement of personal consent for this retrospective analysis was waived by the Ethics Committee (Internal Review Board) of TEDA International Cardiovascular Hospital, so there is no confusion regarding personal consent.

### Date collection

Based on analysis of past studies and expert consensus, as well as convenience of data collection and integrity. This study included the following potential pre-operative risk factors:

1. *Demographic variables*: Sex, age, body mass index (BMI), history of alcohol, history of diabetes mellitus, history of hypertension, history of PCI intervention.
2. *Preoperative drug administration*: β-receptor blocker use, statin use.
3. *Imaging and laboratory examinations*: Last routine blood test/ blood biochemical test(white blood count, red blood count, neutrophile granulocyte to lymphocyte ratio, hemoglobin count, platelet count, albumin to globulin ratio, LDL to HDL ratio, aspartate aminotransferase enzyme to alanine aminotransferase enzyme ratio, creatinine, blood glucose); coronary angiogram and echocardiogram(left main coronary artery stenosis, ejection fraction, ventricular aneurysm, left ventricular end-diastolic diameter, left atrial diameter).

### Definition of POAF

The main endpoint was the incidence of POAF after on-pump CABG, which was defined as the exclusion of paroxysmal or permanent AF by preoperative clinic diagnosis and electrocardiogram, continuous telemetry monitoring or electrocardiogram indicating fibrillatory or absent p waves and its duration lasted more than 10 min before being discharged.

### Statistics

Data was analyzed by SPSS(version 26.0, USA)and Stata(version 17.0, USA). Continuous variables that were not normally distributed were expressed as median ± interquartile, and differences between groups were compared using the Mann–Whitney U test. The continuous variables that were normally distributed were expressed as means ± standard deviation, and differences between groups were compared using the independent test. Categorical variables evaluated by the χ^2^ test were shown as percentage and frequency (%). The association between preoperative LDL/HDL ratio and incidence of POAF was demonstrated by the Lowess curve. Mode I was calculated by logistic regression. The identification of LDL/HDL ratio on POAF was analyzed by receiver operating characteristic (ROC) and area under the curve(AUC). *P* value of < 0.05 was accepted as statistically significant.

## Result

### Demographical characteristics

2052 patients were enrolled in this study, and the incidence of POAF after on-pump CABG was 29.24%. Demographical characteristics were shown in Table 1. In the AF group, statin uses and platelet count were higher (*P* = 0.031, *P* = 0.041, respectively), and LVEDD and LAD were longer (*P* = 0.008, *P* = 0.006). There were no statistical differences among other variables.

**Table 1:** Baseline characteristics between postoperative AF and Non-AF after on-pump CABG

Variables	Non-AF (*n* = 1452)	AF (*n* = 600)	Global cohort (*n* = 1437)	*P*
Demographics				
Sex				0.286
Male	1029 (70.868%)	189 (31.500%)	1440 (70.175%)	
Female	423 (29.132%)	411 (68.500%)	612 (29.825%)	
Age(years)	64.000 (57.000, 69.000)	64.000 (57.000, 68.000)	62.749 ± 8.567	0.449
BMI(kg/m^2^)	25.520 (23.438, 27.736)	25.535 (23.765, 27.757)	25.723 ± 3.185	0.307
History of smoking				0.680
No	883 (60.813%)	359 (59.833%)	1242 (60.526%)	
Yes	569 (39.187%)	241 (40.167%)	810 (39.474%)	
History of PCI				0.213
No	1171 (80.647%)	498 (83.000%)	1669 (81.335%)	
Yes	281 (19.353%)	102 (17.000%)	383 (18.665%)	
Comorbidities				
Diabetes mellitus				0.614
No	903 (62.190%)	366 (61.000%)	1269 (61.842%)	
Yes	549 (37.810%)	234 (39.000%)	783 (38.158%)	
Hypertension				0.332
No	427 (29.408%)	187 (31.167%)	614 (29.922%)	
Grade 1	86 (5.923%)	24 (4.000%)	110 (5.361%)	
Grade 2	308 (21.212%)	130 (21.667%)	438 (21.345%)	
Grade 3	631 (43.457%)	259 (43.167%)	890 (43.372%)	
Preoperative drug use				
β-Receptor blocker				0.504
No	359 (24.725%)	140 (23.333%)	499 (24.318%)	
Yes	1093 (75.275%)	460 (76.667%)	1553 (75.682%)	
Statin				0.031
No	117 (8.058%)	32 (5.333%)	149 (7.261%)	
Yes	1335 (91.942%)	568 (94.667%)	1903 (92.739%)	
ACEI/ARB				0.782
No	994 (68.457%)	407 (67.833%)	1401 (68.275%)	
Yes	458 (31.543%)	193 (32.167%)	651 (31.725%)	
Examination				
White blood count(× 10^9^/L)	6.300 (5.400, 7.500)	6.400 (5.300, 7.500)	6.535 ± 1.675	0.716
Red blood count(× 10^12^/L)	4.400 (4.000, 4.800)	4.400 (4.100, 4.700)	4.419 ± 0.998	0.575
NLR	2.089 (1.568, 2.825)	2.095 (1.531, 2.720)	2.397 ± 1.502	0.594
Hemoglobin (g/ dl)	135.000 (123.000, 147.000)	136.000 (124.000, 147.000)	135.021 ± 18.501	0.497
Platelet count(× 10^9^/L)	214.000 (180.000, 253.000)	219.500 (181.750, 262.000)	220.306 ± 58.281	0.041
A/G	1.615 (1.448, 1.800)	1.600 (1.448, 1.792)	1.629 ± 0.292	0.837
AST/ALT	0.904 (0.694, 1.176)	0.895 (0.694, 1.154)	1.039 ± 0.802	0.614
Creatinine (mg/ dl)	69.000 (59.000, 81.000)	69.000 (58.000, 80.000)	72.245 ± 33.236	0.331
LDL(mmol/L)	2.545 (1.970,3.300)	2.610 (1.980,3.340)	2.748 ± 0.991	0.236
HDL(mmol/L)	0.990 (0.850,1.150)	0.990 (0.840,1.140)	1.022 ± 0.621	0.538
LDL/HDL	2.630 (2.030,3.350)	2.670 (2.138,3.360)	2.766 ± 0.996	0.115
Left main coronary artery stenosis				
No	1067 (73.994%)	441 (74.118%)	1508 (74.030%)	
Yes	375 (26.006%)	154 (25.882%)	529 (25.970%)	
EF (%)	61.000 (55.000, 65.000)	61.500 (56.000, 66.000)	59.387 ± 9.491	0.138
LVEDD(mm)	48.000 (45.000, 51.000)	48.000 (46.000, 52.000)	48.841 ± 5.514	0.008
LAD(mm)	37.000 (35.000, 40.000)	38.000 (35.000, 41.000)	38.058 ± 5.307	0.006

BMI: body mass index; PCI: percutaneous coronary intervention; ACEI: Angiotensin-Converting Enzyme Inhibitors; ARB: Angiotensin Receptor Blockers; NLR: neutrophile granulocyte to lymphocyte ratio; A/G: albumin to globulin ratio; AST/ALT: aspartate aminotransferase enzyme to alanine aminotransferase enzyme ratio; EF:ejection fraction; LVEDD: left ventricular end-diastolic diameter; LAD: left atrial diameter

### Lowess curve analysis

**Fig. 1:**
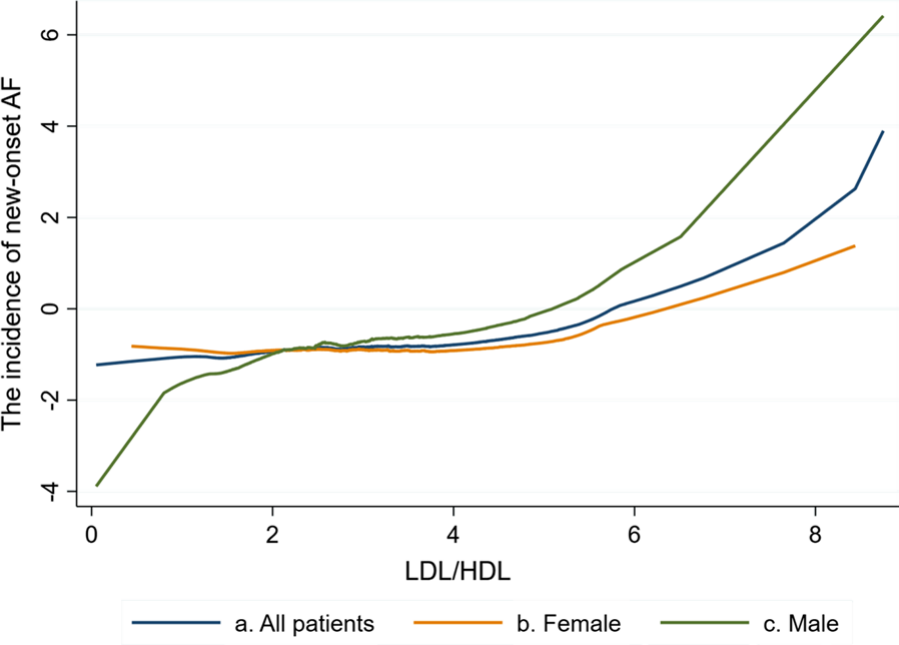
Association between LDL/HDL and POAF after on-pump CABG. Curve a, the association between LDL/HDL and POAF after on-pump CABG in all patients. Curve b, the association between LDL/HDL and POAF after on-pump CABG in females. Curve c, the association between LDL/HDL and POAF after on-pump CABG in males

The association between preoperative LDL/HDL ratio and POAF after on-pump CABG was similar to a linear relationship (Fig. 1a), with the increasement of preoperative LDL/HDL, the incidence of POAF increased simultaneously. POAF was affected by Sex, and thus Lowess curve was recalculated in different sex groups. As shown in the tables, the association between preoperative LDL/HDL and POAF after on-pump CABG in Female (Fig. 1b) and male (Fig. 1c) were also similar to a linear relationship.

### Logistic regression analysis

Univariate logistic regression analysis revealed that preoperative LDL/HDL ratio was directly related to POAF (*OR* = 1.13, 95% CI = 1.03–1.24, *P* = 0.012); Among female patients, *OR* is 1.345 (95%CI = 1.136–1.593, *P* = 0.001). Among male patients, *OR* is 1.037(95% CI = 0.924–1.164, *P* = 0.536). Among female patients, preoperative LDL/HDL ratio was still directly related to POAF after adjustment of age and MBI (Model I) (*OR* = 1.340,95% CI = 1.131–1.588, *P* = 0.001); After adjusting age, BMI, preoperative statin administration, platelet count, LVEDD and LAD (Model II), preoperative LDL/HDL ratio was still a predictor of POAF after on-pump CABG (*OR* = 1.306, 95% CI = 1.100–1.550, *P* = 0.002) (Table 2).

**Table 2:** Adjusted *OR* values

Variables	*OR*	95%*CI* of *OR*		*P*
		Lower limit	Upper limit	
*Model I*				
LDL/HDL	1.340	1.131	1.588	0.001
Age(years)	0.987	0.964	1.011	0.302
BMI(kg/m^2^)	1.027	0.973	1.084	0.337
*Model II*				
LDL/HDL	1.306	1.100	1.550	0.002
Age(years)	0.989	0.965	1.013	0.374
BMI (kg/m^2^)	1.031	0.976	1.089	0.276
Statin	1.538	0.706	3.348	0.278
Platelet count(× 10^9^/L)	1.002	0.999	1.005	0.112
LVEDD (mm)	0.990	0.951	1.029	0.604
LAD (mm)	1.041	0.997	1.087	0.068

Adjustments of Model I were age and BMI: body mass index LVEDD; Adjustment of Model II were age, BMI: body mass index; preoperative Statin took; Platelet count; LVEDD: left ventricular end-diastolic diameter; LAD: left atrial diameter

### ROC curve analysis

**Fig. 2:**
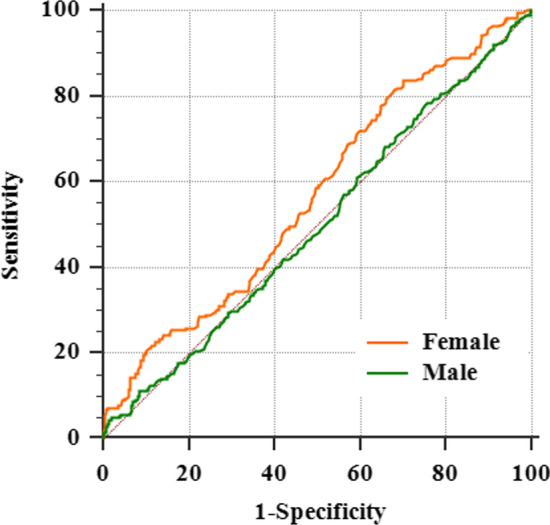
ROC curve analysis of LDL/HDL to predict POAF after on-pump CABG

As shown in Fig. 2, preoperative LDL/HDL could identify postoperative arrhythmia after on-pump CABG (AUC = 0.569, 95% CI = 0.529–0.608, *P* = 0.006) among female patients, the best preoperative LDL/HDL ratio cutoff of 2.11, which was considered a predictive factor of incident POAF, showed a sensitivity of 83.60% (95% CI = 0.775–0.886) and a specificity of 30.02% (95% CI = 0.257–0.346). There was no statistically significant difference in preoperative LDL/HDL to predict incident POAF among male patients (AUC = 0.500, 95% CI = 0.474–0.527, *P* = 0.981).

**Fig. 3:**
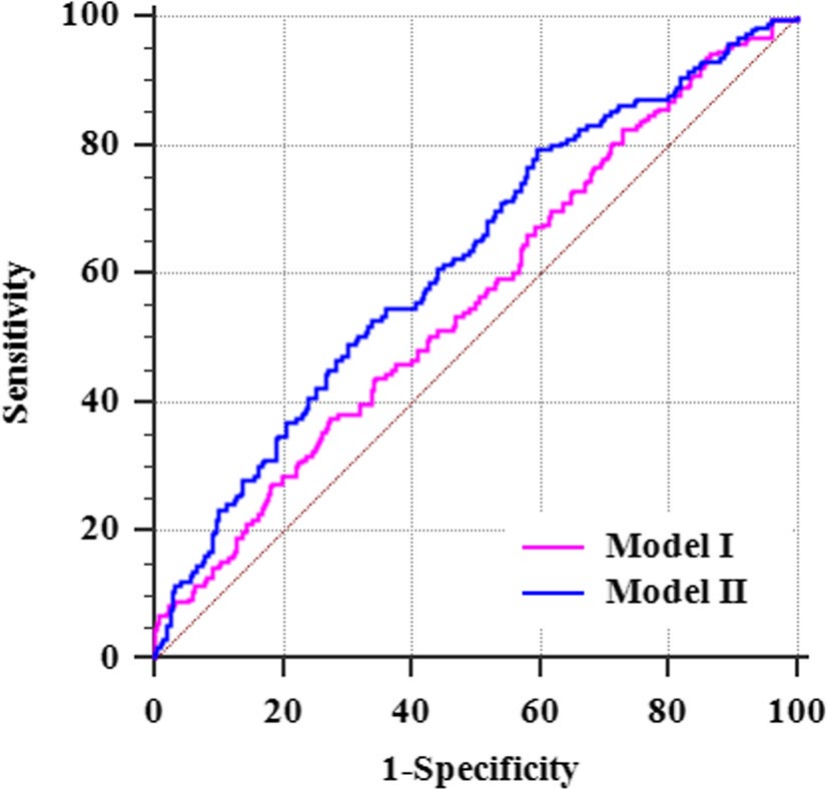
ROC curve analysis of Model I and Model II

As shown in Fig. 3, AUC of Model I to predict incident POAF in female patients was found to be 0.562 (95% CI = 0.522–0.602, *P* = 0.013), predictive potence more than 11.20% showed a sensitivity of 37.57% (95% CI = 0.306–0.449) and a specificity of 72.58% (95% CI = 0.681–0.768); AUC of Model II to predict incident POAF in female patients was 0.622 (95% CI = 0.583–0.661), predictive potence more than 26.16% showed a sensitivity of 79.37% (95% CI = 0.729–0.849, *P* < 0.001) and a specificity of 40.43% (95% CI = 95% CI = 0.357–0.453).

## Discussion

The pathogenesis of new-onset POAF is involved in multi-factor and multi-mechanism. In the present study, both groups have no significant differences in demographical characteristics before surgery. The total incidence of POAF is 29.24%, consistent with researches at home and abroad [16, 17].

The underline pathogenesis of POAF included pre-existing degenerative changes in the atrial myocardium and abnormalities of electrophysiologic parameters. A large bunch of clinic studies have been published to identify the risk factors related to POAF. The previous researches have demonstrated that age, obesity, race, previous cardiac surgery and so on were associated with POAF [18–20]. Additionally, a great many of models were built to predict POAF, with which doctors will make a quicker response for some severe complication [21–23].

Dyslipidemia is considered as the main cause of coronary atherosclerotic heart disease, which changes cell membrane content and electrophysiological property. In China, the standard lipid level(TC, LDL-C,HDL-C,TG) are of < 5.2 mmol/L, < 3.4 mmol/L, > 1 mmol/L and < 1.7 mmol/L, respectively. The Changes in membrane cholesterol content can change Ion channel distribution and functional properties of the membrane, inducing AF [24]. Intense studies have demonstrated the relationship between LDL and(or) HDL and AF, with discrepancies in different studies. Investigation in the Chinese hypertension population demonstrated that low LDL level was related to increased incident AF [25]. The same result was also demonstrated in a meta-analysis [26]. Turkkolu et al. proved that low LDL level was related to increased incident POAF in patients after CABG [27], whereas another retrospective study showed a different result [28]. Different from the vascular endothelial injury caused by LDL, HDL has anti-inflammatory, anti-oxidative, and anti-thrombotic properties which also plays an important role in the pathogenesis and development of AF [5, 29]. The research published by Okin et al. indicated a strong correlation between LDL level and AF [30]. A meta-analysis evidenced the direct correlation between high HDL level and low incident AF [26]. Tekkesin et al. used a new oxidative indicator to predict POAF after CABG, the result suggested that Monocyte to HDL (M/H) ratio was found to be statistically significantly higher in POAF( +) patients than in POAF(−) patients [31]. M/H is also a valuable predictor of early recurrence of AF after valvular repair surgery [32].

LDL/HDL ratio has been suggested as a predictor of severity of atherosclerosis, graft patency after CABG, and carotid artery intima-media thickness because it influenced the occurrence and development of AF [10, 33–35], whilst investigations about preoperative LDL/DHL ratio and new-onset POAF are seldom. In the present study, the Lowess curve showed that the association between preoperative LDL/HDL ratio and POAF after on-pump CABG was similar to a linear relationship, as well as in different sex groups. Univariate logistic regression analysis revealed that the preoperative LDL/HDL ratio was directly related to POAF (OR > 1, *P* < 0.05). In female patients, preoperative LDL/HDL ratio was associated with incident POAF after on-pump CABG(OR > 1, *P* < 0.05). However, no statistical significance was found in male patients. This result can be partly explained by differences on the history of smoking, drinking, and hormone level which plays a vital role in lipid metabolism. Kim et al. thought lipoprotein in men could exhibit strong pro-inflammatory and pro-atherogenic properties [36]. Thus, the preoperative LDL/HDL ratio may not be the main cause for PAOF, further analysis is needed to explore possible mechanisms between sex and POAF after CABG.

ROC analysis showed that preoperative LDL/HDL ratio could identify postoperative arrhythmia (AF or sinus rhythm) after on-pump CABG (AUC = 0.569, 95% CI = 0.529–0.608, *P* = 0.006) among female patients. the best preoperative LDL/HDL ratio cutoff of 2.11, which was considered a predictive factor of incident POAF, showed a high sensitivity of 83.60% (95%CI = 0.775–0.886) and a low specificity of 30.02% (95%CI = 0.257–0.346). So LDL/HDL ( +) can predict incident POAF with a possible misdiagnosis rate. Results from Model II proved that multiple model prediction can strengthen the identification of preoperative LDL/HDL on postoperative rhythm and increase its specificity to avoid incorrect diagnosis. This indicated that LDL/HDL was associated with incident POAF among female patients suggesting that more lipid-related indices might be enrolled together to build a more efficient prediction model in the future.

There are some limitations in our study. This is a single-center retrospective study. Although we enrolled consecutive patients there are still biases in our study. Further multicenter prospective researches with the long-term following are needed to demonstrate the relationship between preoperative LDL/HDL ratio as well as other lipid indices and long-term POAF.

## Conclusion

Above all, the association between preoperative LDL/HDL ratio and POAF after on-pump CABG was similar to a linear relationship**.** but there is a difference in different sex. Preoperative LDL/HDL level can help to identify postoperative rhythm in females. In females, a preoperative LDL/HDL level of more than 2.11 should alert the surgeon to the possibility of POAF.

## Data Availability

All data generated or analyzed during this study are included in this published article.

## Abbreviations

PCI: Percutaneous coronary intervention
CABG: Coronary artery bypass grafting
AF: Atrial fibrillation
POAF: Postoperative atrial fibrillation
LDL: Low density lipoprotein
HDL: High density lipoprotein
BMI: Body mass index
LVEDD: Left ventricular end-diastolic diameter
LAD: Left atrial diameter
OR: Odd ratio
ROC: Receiver operating characteristic
AUC: Area under the curve
M/H: Monocyte to HDL
